# Best practices for authors of healthcare-related artificial intelligence manuscripts

**DOI:** 10.1038/s41746-020-00336-w

**Published:** 2020-10-16

**Authors:** Sujay Kakarmath, Andre Esteva, Rima Arnaout, Hugh Harvey, Santosh Kumar, Evan Muse, Feng Dong, Leia Wedlund, Joseph Kvedar

**Affiliations:** 1 MGH & BWH Center for Clinical Data Science, Partners Healthcare, Boston, MA USA; 2 Department of Medical AI, Salesforce Research, Palo Alto, CA USA; 3 Division of Cardiology and Bakar Computational Health Sciences Institute, University of California, San Francisco, CA USA; 4 Hardian Health, London, UK; 5 The University of Memphis, Memphis, TN USA; 6 Scripps Research Translational Institute, La Jolla, CA USA; 7 Human Centric AI Research Group, University of Strathclyde, Glasgow, Scotland; 8 Harvard Medical School, Boston, MA USA; 9 Partners HealthCare, Boston, MA USA

**Keywords:** Diagnosis, Publication characteristics, Outcomes research

## Abstract

Since its inception in 2017, *npj Digital Medicine* has attracted a disproportionate number of manuscripts reporting on uses of artificial intelligence. This field has matured rapidly in the past several years. There was initial fascination with the algorithms themselves (machine learning, deep learning, convoluted neural networks) and the use of these algorithms to make predictions that often surpassed prevailing benchmarks. As the discipline has matured, individuals have called attention to aberrancies in the output of these algorithms. In particular, criticisms have been widely circulated that algorithmically developed models may have limited generalizability due to overfitting to the training data and may systematically perpetuate various forms of biases inherent in the training data, including race, gender, age, and health state or fitness level (Challen et al. BMJ Qual. Saf. 28:231–237, 2019; O’neil. Weapons of Math Destruction: How Big Data Increases Inequality and Threatens Democracy, Broadway Book, 2016). Given our interest in publishing the highest quality papers and the growing volume of submissions using AI algorithms, we offer a list of criteria that authors should consider before submitting papers to *npj Digital Medicine*.

Others have published guidelines for manuscript submissions as well. While there is some overlap there are important differences. One key theme we hope to highlight in these guidelines is that *npj Digital Medicine* is a journal focused on innovation in digital medicine. As such we encourage authors to justify their choice of machine learning algorithms in the context of a clinical problem and clarify their methodological innovations.

In this editorial, we will lay out a series of priorities and considerations for submitting authors. First and foremost amongst these recommendations is choosing a topic and problem that has a clear health context. The model you create should have a clear diagnostic or prognostic relationship to an important health problem and there should be some explanation of how the strengths/limitations of existing models supported development of a new project.

## In order to qualify for submission to *npj Digital Medicine*, the innovation should also be a digital medicine innovation

Contributions outside of digital medicine—e.g., genetics, molecular, cardiac, radiology, etc.—that merely utilize machine learning algorithms on traditional data without justifying how such an application might add value given the status quo, should be sent to their respective specialty journals. Digital medicine innovations should provide some potential clinical benefit beyond the status quo in the realm of either diagnosis or treatment.

## The datasets used for model development, validation, and testing should be adequately described

Describe the digital datasets used for training, validation, and testing, including any differences between these datasets^3^. A separate test dataset external to the ones used for model development and validation must be used to assess and report the final model performance. Include measures taken to ensure that the data in the test set and the training/validation sets are independent of each other (e.g., zero overlap between training and test sets). Overlap between training and test datasets could artificially inflate test set performance. Samples within a dataset that are interdependent (e.g., multiple pictures of the same skin lesion, from different angles) should be disclosed, contained within a single subset (e.g., training), and not split across train/validation/test sets. Provide definitions, methods and relevant context for the input data variables and the output variables of the AI task(s), including justifications for any modifications made to the original data (e.g., changing continuous data to the discrete, exclusion of certain data points, handling of missing data, and so on)^4^. Describe what ground-truth label was used, why it was chosen, and its relationship to the clinical gold-standard where applicable. If labels are assigned by human experts, describe methods in detail. Describe any efforts to quantify, and mitigate, intra- and inter-observer labeling differences^5^. Also, describe how closely the temporal alignment of the labels relates to the data segments being assigned. Include any methodology used in pre-processing, post-processing, or otherwise altering the data, and how this would be done if deployed. Each dataset should be diverse in demographic and other relevant dimensions (e.g., vendor type) to allow for broad generalizability^2^. Explain why the test set is a representative sample and allows you to conclude the claims of the paper. Describe biases it may contain, and ethical considerations that could arise as a result of this bias^6,7^. Justify the sample size of the dataset; potential ways to justify sample size may include: statistical guidance^8^, comparison with sample size used in previous studies describing analogous models, empirical assessments of model performance by relative sample size, error bar analysis, using re-sampling techniques such as bootstrap sampling^9^, characterizations of out-of-distribution samples in the test set, or sufficiency of the sample size via model performance saturation with increase in the size of input data. Also identify and report limitations of the dataset relevant to the context of the problem (representativeness, bias, measurement error)^10^.

## Provide a detailed description of the methods used for model development and testing

First describe why a pattern to be identified by the model from the data is to be expected given current knowledge in the domain science. Describe the outcome to be predicted by the model (for example, the model classifies the presence or absence of a fracture on wrist X-rays). Describe different modeling choices and justification of the models eventually selected for comparisons. Specify the type of models and describe all model building procedures for replication studies. This should include: detailed description of the model architecture (inputs, outputs, filter sizes, layers, and cost functions), details of training approach, including data augmentation steps and parameters, network hyperparameters, number of models trained, regularization methods, and the process used to select final models, and descriptions of how weight parameters were initialized. (e.g., random or drawn from a particular distribution). Also, describe method and metrics used for internal validation of the model, as well as those used to guide parameter selection. Include the steps taken to avoid and assess overfitting, such as testing of the trained model on an independent dataset of comparable size to the training dataset^11^. Discuss the types of initialization methods used, if relevant, for any models.

## Describe the model’s performance

Report all performance metrics with confidence intervals on validation and test datasets and report model calibration where applicable^6^. Compare performance with existing models, if possible^12^. If baseline methods are used for model comparison, explain why they are fair methods to compare against yours. If possible and reasonable, report results both in the context of model performance metrics (e.g., Dice, F-score, etc) and of clinical performance metrics (sensitivity, number needed to treat, etc)^13^. If possible and reasonable, benchmark against human performance. If possible and relevant, report false positive rates per time unit (e.g., per day, per week, etc.), instead of per data point, given wide variability in the length of data that may be used as an input unit. All comparisons of model performance (with humans; against other models, etc) need to be backed by statistics.

## Discuss the limitations of the model and/or the methods used

Describe how the robustness of the model was assessed and report any results from such experiments^14^. Address potential challenges involved in scaling data collection or applying the model to existing datasets. If the dataset and source code of the model are publicly available, guidelines for citation of publicly available datasets can be found at: https://www.nature.com/documents/nr-data-availability-statements-data-citations.pdf. Clinical trials involving the use of machine learning-based solutions should report in accordance with CONSORT guidelines^15^.

## Describe the proposed clinical context and workflow with model implementation (a schematic diagram is recommended)^16^

### **Discuss the implications of errors made by the model on clinical and economic outcomes**

If the manuscript addresses potential cost-savings or quantitative clinical benefits, please provide sensitivity analyses. Also discuss and present failure cases and analysis of these failures.

### **Describe the generalizability of the model**,

Including the performance of the model on validation and testing datasets. Clarify whether transfer learning is applied to the model training and where applicable present details of the transfer learning process. Discuss the transferability of the model to other clinical cases

### **Present clinical acceptability and user perceptions**

Describe the model’s pertinence to humans. Where appropriate, report user perceptions on the models and their outputs, and describe the trustworthiness of the models. Where appropriate, also describe the integration of the models to clinical workflows.

Our hope is that these guidelines and best practices will help authors innovating in the area of digital medicine to focus their research and manuscripts. A keen sense of clinical applications, combined with a standardized discussion of methods and performance metrics may help us raise the quality of contributions in the field.
